# Thoughts and Behaviors of Chinese and Japanese Doctors when faced with the Death of a Patient: A Qualitative Descriptive Study of Doctors’ Responses to a Hypothetical Scenario

**DOI:** 10.1007/s41649-024-00326-0

**Published:** 2025-04-02

**Authors:** Hua Xu, Taketoshi Okita, Masao Tabata, Yasuhiro Kadooka, Atsushi Asai

**Affiliations:** 1 Department of Medical Ethics, Tohoku University Graduate School of Medicine, Sendai, Japan; 2 Division of Philosophy and Ethics, Department of Culture and Medicine, School of Medicine, Shiga University of Medical Science, Otsu, Japan; 3 Patient Safety Management Office, Tohoku University Hospital, Sendai, Japan; 4 Department of Bioethics, Faculty of Life Sciences, Kumamoto University Graduate School of Medicine, Kumamoto, Japan

**Keywords:** Qualitative descriptive analysis, Cross-cultural comparison, Error disclosure, Apology, China, Japan

## Abstract

China and Japan have similar cultures but differing healthcare systems. In both countries, admissions of medical error and apologies by doctors continue to be an important but difficult issue. The present study aimed to examine and compare the thoughts and behaviors of Chinese and Japanese doctors when faced with the unexpected death of a patient. Qualitative descriptive analysis was performed to compare the responses of 20 doctors from each country to a hypothetical scenario involving the death of a patient. We found that almost all doctors in both countries considered the treatment process described in the hypothetical scenario to be inappropriate and most would feel regret when faced with the young patient’s death. There was a disagreement concerning responsibility for the patient death among the doctors regardless of their nationality. Doctors decided how to behave facing the patient death after anticipating the bereaved family’s reaction and their initial responses varied widely. Japanese doctors indicated that they would communicate with the patient’s family after a patient died, whereas none of the Chinese doctors indicated they would do so due to a fear of physical violence from the bereaved family. Finally, the decision on whether to disclose the medical error and apology was made after careful and complex consideration. In conclusion, significant differences were observed between Chinese and Japanese doctors with respect to communicating with, and disclosing errors and apologizing to, the bereaved family. We discuss both the ethical and social implications of these differences.

## Introduction

A harmonious doctor-patient relationship (DPR) is ethically important and clinically invaluable to patients, their families, and doctors. The DPR involves considerable intimacy, vulnerability, and trust (Robbennolt 2009). However, inappropriate attitudes of doctors towards patients and inadequate information disclosure when unanticipated adverse events occur, especially when medical errors might be involved, have a strong negative impact on DPRs, particularly on patients’ trust in doctors and healthcare organizations (Citizens’ Association for the Disclosure and Publication of Medical Information et al. 2021).

An adverse event refers to an injury that was caused by medical management rather than the patient’s underlying disease, and a medical error refers to the failure of a planned action to be completed as intended or the use of a wrong plan to achieve an aim (Gallagher et al. 2006). The need for doctors to be sincere in their attitudes and behaviors when adverse events occur due to medical errors has long been emphasized (Lazare 2006; Wachter and Kiran 2018; Nagai 2019; Fukuzaki 2021; Wakamatsu 2023). From an ethical perspective as well, fundamental respect for patients requires not only that they be informed of the error and its consequences but also that an apology be offered for any harm done. While the inclination to conceal medical errors must be discouraged (Jonsen et al. 2015), existing reports from various countries suggest a difference between the attitudes and behaviors of doctors and the ethics advocated when medical errors occur in patient care. For generations, many doctors and healthcare systems have responded with silence after medical errors occur (Gallagher et al. 2006; Wachter and Kiran 2018; Mendonca et al. 2019). While the ethical approach would be for doctors to admit to their medical errors, the instinct of doctors is to do the opposite (Detsky et al. 2013). A healthcare environment and culture that enable doctors to offer honest apologies are important. Yet, serious obstacles exist to realize this practice, including the fear of lawsuits and damage to one’s reputation (McLennan et al. 2014; Loue 2020).

Although adverse events resulting from medical errors occur in both China and Japan, to the best of our knowledge, few empirical studies have examined the attitudes and behaviors of doctors regarding error disclosure and apologies when faced with such errors. Anecdotal case reports by patients or the bereaved family who lost their loved one due to medical errors as well as newspaper articles have long criticized the concealment of medical errors and dishonest disclosure by doctors and medical institutions (Gao et al. 2015; Citizens’ Association for the Disclosure and Publication of Medical Information et al. 2021).

Given the scarcity of studies on the thoughts and behaviors of Chinese and Japanese doctors regarding admitting to medical errors and apologizing, we conducted an exploratory qualitative descriptive study targeting these doctors using a hypothetical scenario (Table 1) to better understand how they react when faced with an unanticipated adverse event that results in a patient’s death, possibly due to a medical error. Revealing the attitudes and behaviors of doctors on this topic is the initial step to addressing issues of disclosure and apologies for medical errors in both countries. If the major obstacles to admitting to errors and offering apologies can be identified, then realistic and comprehensive countermeasures can be developed, leading to improvements in the wellbeing of patients and their satisfaction with healthcare and DPRs.

**Table 1:** Hypothetical scenario

A 28-year-old male was involved in a fight and was transported to the hospital one hour after injury for severe trauma to the body, including the head, and a knife wound to the neck. After admission, Doctor X provided the man with the treatment he deemed necessary but only performed debridement and suturing of the neck wound and did not adequately examine the depth of the neck puncture wound or the presence of cervical vascular injury. Fifteen hours after admission, the patient began to complain of dyspnea. After the patient developed a severe cough, his neck suddenly swelled, suggesting massive internal bleeding. Three hours later, or 18 h after admission, the patient died despite emergency care by Doctor X. The patient’s family was surprised by the patient’s death and seemed totally unable to accept the patient’s death and appeared to be angry.	

China and Japan have similar cultures but differing healthcare systems. Comparative research of China and Japan is informative for a number of reasons (Xu et al. 2024). First, a comparative study can identify factors that are strongly related to the doctor’s attitudes towards and views of admission of medical errors and apologies as there are similarities and differences between the two countries in terms of the healthcare system and medical environment. Second, our comparative study can demonstrate how differences in cultural and social aspects, interpersonal differences, and historical changes in the two countries are related to admission of medical errors and apologies. Third, experiences and perceptions of doctors from both countries can provide useful information for people of both countries to learn about and from each other. For instance, results from such studies may provide insight into means for establishing clinical guidelines concerning admission of errors and apologies in each country. Finally, it is also expected that there will be more opportunities to access each other’s medical systems from citizen-level exchanges between China and Japan. Therefore, we think that it is necessary to deepen our understanding by comparing the current state of medical care in both countries (Xu et al. 2024).

## Methods

The present study is part of a China-Japan comparative study on doctor-patient relations, doctor-patient disputes, and doctor well-being. In the main study using the same questions, semi-structured interviews were conducted with 20 Chinese doctors and 20 Japanese doctors regarding doctor-patient relationships, decision-making models, current state of medical disputes, and doctor well-being in both countries as well as their attitudes toward and thoughts about a hypothetical scenario involving the death of young man. Doctors in both countries were sampled through the researchers’ personal networks. Snowball sampling was also used for some participants. All participating doctors were currently working doctors who had already completed their clinical training by the time of the interviews. Interviews were conducted in both countries over a two-month period spanning March and April 2023.

This study was approved by the Ethics Committee of Tohoku University Graduate School of Medicine on January 26, 2023 (2022-1-886). At the time of conducting the interviews, the method and intent of the study were explained, and all participants provided written informed consent. All participants were informed that the contents of the interviews would be recorded and that their statements would be reported anonymously. No honorarium was paid for participating in the study. Qualitative descriptive analysis was used to identify doctors’ thoughts and actions when faced with the death of a patient involved in a hypothetical scenario (Table 1) in a qualitative and inductive manner in the form of subcategories and categories (Sandelowski 2000; Satu and Kyngas 2008; Gibbs 2018).

This hypothetical scenario was used for the following reasons. First, the characteristics of the scenario and its outcome are very common in clinical practice in both China and Japan. Since this is a cross-national comparative study, the scenario used should be one that is common in both countries. Second, one of the authors experienced a similar situation involving the death of a young patient as a doctor and was involved in the subsequent medical dispute. Therefore, responses of the participating doctors to this hypothetical scenario can provide realistic and relevant insights into the medical, political, and social environments and cultures of both countries. Third, a patient’s sudden death is one of the worst scenarios that doctors face. Responses to the aftermath of the hypothetical scenario could shed light on what doctors from both countries would think, feel, and do in worst-case scenarios. Finally, hypothetical scenarios have been used to examine doctor’s attitudes towards and behaviors regarding error disclosure and apologies (Gallagher et al. 2006).

According to Sandelowski, qualitative description is a complete and valued end-product in itself rather than an entry point into other qualitative studies such as grounded theory, phenomenology, or ethnography, and qualitative descriptive studies have a long history in academic fields (Sandelowski 2000). Qualitative descriptive studies can offer a comprehensive summary of an event in everyday terms of those events and are the methods of choice when straight descriptions of phenomena are desired and the study method is amenable to obtaining straight and largely unadorned answers to questions of special relevance to practitioners and policy makers, including questions concerning people’s concerns, responses, reasons, and influencing factors on a given issue (Sandelowski 2000). Qualitative description is less interpretative than the above-mentioned methods and does not require a conceptual or other otherwise highly abstract rendering of data (Sandelowski 2000). Researchers conducting qualitative descriptive studies stay closer to their data; qualitative descriptive studies may be the least theoretical of the spectrum of qualitative approaches and are the least encumbered by pre-existing theoretical and philosophical commitments (Sandelowski 2000). However, qualitative descriptive studies are not atheoretical, and they tend to follow the philosophical foundations of natural inquiry, usually describing a participant’s experience directly or presenting an event in simple language as free of artifice as possible in the artifice-laden enterprise known as conducting research (Ma et al. 2023; Sandelowski 2000, 2010).

The initial steps of the qualitative descriptive analysis were conducted by Hua Xu (H.X.) and Atsushi Asai (A.A.), who then shared their results with all researchers/authors and discussed them until a consensus was reached on the contents. More specifically, the following steps were taken. First, from the verbatim transcripts of each interview, the responses of participating doctors to the following three questions concerning the hypothetical scenario (Table 1) were extracted: (1) What do you think of the outcome of the scenario? (2) If you were this patient’s doctor, how would you handle this situation? (3) What is your attitude towards, and what do you talk about with, the patient’s bereaved family? Second, the researchers (H.X., A.A.) reviewed the responses to these questions several times to obtain a complete picture of the interviewees’ responses. Third, meaning units related to the responses were listed in two Excel files (one each for Chinese and Japanese doctors), followed by coding of the meaning units in each file. Codes with similar content were grouped into subcategories, and subcategories with common themes or implications were grouped into categories. We continued the same process iteratively and all researchers confirmed and agreed on the appropriateness of all generated codes, subcategories, and categories. “CD” refers to a Chinese doctor, and “JD” to a Japanese doctor. Relevant portions regarding the thoughts, feelings, or attitudes of participating doctors are provided in direct quotes.

During the analysis, all researchers, including four (H.X., A.A., T.O., and Y.K.) with experience conducting qualitative research, engaged in discussions to ensure the consistency, validity, and reliability of data. Based on previous studies, the target sample size of cross-cultural studies to reach theoretical saturation ranges from 12 and 30 (Alshahrani et al. 2022). In the present study, 40 doctors were interviewed. In the qualitative descriptive analysis of verbatim interview transcripts of 20 Chinese and 20 Japanese doctors, by the end of analyzing 20 interviews each, no new reasons for medical conflicts were raised, and thus new codes and subcategories were unlikely to be generated by increasing the size of the study population.

## Results

The background of participating doctors is shown in Table 2. The qualitative descriptive analysis identified five categories and nineteen subcategories, which are shown in Table 3.

**Table 2:** Demographic characteristic of the study participants

Category	China	Japan
	No. (%) or mean ± SD (*N* = 20)	No. (%) or mean ± SD (*N* = 20)
Interview time mean (min)	50 (27–148)	52 (37–80)
Gender		
Female	4 (20)	3 (15)
Male	16 (80)	17 (85)
Age		
20 s	1 (5)	0
30 s	4 (20)	1 (5)
40 s	12 (60)	5 (25)
≧ 50 s	3 (15)	14 (70)
Working experience	16 ± 8	29 ± 13
5–9 years	4 (20)	0
10–19 years	12 (60)	2 (10)
20–29 years	2 (10)	9 (45)
≧30 years	2 (10)	9 (45)
Hospital type		
Primary healthcare institution		5 (25)
Secondary hospital		6 (30)
University hospital	20 (100)	9 (45)
Specialty		
Internal medicine	2 (10)	14 (70)
ER	3 (15)	
Surgery	14 (70)	6 (30)
ICU	1 (5)	
Conflict experience with patient and/or its family		
Aggressive attitudes/words	19 (95)	2 (10)
Physical attack	6 (30)	1 (5)
Personal/professional involvement in formal dispute process	17 (85)	7 (35)

**Table 3:** Responses of Chinese and Japanese doctors to hypothetical scenario

Categories (*N* = 5)	Subcategories(*N* = 19)
Most doctors considered the treatment process to be inappropriate	The doctor was inexperienced and lacked adequate skills (CD and JD)
	Medical errors occurred during treatment (CD and JD)
	The death was related to the quality of the hospital, workload, and lack of contingency plans (CD and JD)
Doctors felt regret about the young patient’s death	I would feel regret for the patient’s death (CD and JD)
	Both the doctor and patient were unlucky (CD)
	The more you experience some sudden death scenarios, the more timid you become (CD)
There was a disagreement concerning responsibility for the patient’s death among doctors from both countries	If there was no medical error, the doctor should be exempt from a lawsuit (CD)
	The fight brought about the patient death (CD and JD)
	It is inevitable for the family to be angry and initiate a lawsuit (CD and JD)
	This case is very complicated and requires discussion concerning responsibility for the patient’s death (CD and JD)
Participating doctors decided how to behave after anticipating the reaction of the bereaved family	I would have reported to the patient safety department of my hospital first when the patient died (CD and JD)
	It is impossible for doctors to communicate with the family due to the possibility of physical violence from bereaved family members (CD)
	I need to communicate with the family because they would find me quickly (CD)
	The first major premise is to properly explain what happened to the family (JD)
	It depends on the situation such as the treatment process, characteristics of the patient’s family, and hospital setting (JD)
The decision on whether to disclose an error and apologize to the bereaved family was made after careful and complex consideration	I would not admit to my error to family members without an expert’s investigation into the cause of the patient’s death to avoid unnecessary trouble (CD and JD)
	I would apologize to the patient’s family if I thought that I had made a medical error when providing patient care (CD and JD)
	The magnitude of the medical error changes doctor’s attitudes towards honest disclosure (CD)
	If there was a medical error, it should be explained to the family as soon as possible to avoid a situation where the family suspects the hospital is hiding the error (JD)

“CD” indicates Chinese doctor, and “JD” indicates Japanese doctor

### Most Doctors Considered the Treatment Process to be Inappropriate

#### The Doctor was Inexperienced and Lacked Adequate Skills (CD and JD)

Doctors from both countries noted the inexperience and lack of skills of Doctor X in the hypothetical scenario. Most participating doctors, irrespective of their nationality, stated that Doctor X lacked diagnostic competence, failed to explain the patient’s condition in advance, did not perform an adequate examination and follow-up, and failed to consult with a senior doctor or other specialist in a timely manner. Several doctors stated that this was a very common treatment scenario, and that Doctor X should have anticipated major hemorrhage and sudden death when treating the patient.

“This doctor with poor skills should have asked for help from a senior doctor.” [CD13]

“Neck anatomy is complex and thus it is better to examine the neck more carefully than to treat only the wound.” [JD2]

#### Medical Errors Occurred during Treatment (CD and JD)

Many doctors from both countries believed that there were medical errors in Doctor X’s treatment process and that the patient died from these errors. Doctors noted that the manner in which the patient’s wounds was assessed is problematic and that a diagnostic error had been made. It was also pointed out that patients are sometimes treated by technically and mentally inexperienced doctors like Doctor X. Several doctors stated that if they had been the doctor in charge, they would have treated the patient differently and that 18 h would have been sufficient to save the patient’s life.

“It’s the doctor’s error.” [CD10]

“I think that it was an error. The important site of injury should be given priority and observed carefully.” [JD17]

#### The Death was related to the Quality of the Hospital, Workload, and Lack of Contingency Plans (CD and JD)

Doctors from both countries believed that the size of the hospital, its clinical quality and busyness, whether it had an emergency department or intensive care unit, and whether it had contingency plans played roles in the patient’s death. Some doctors commented that in a university hospital with multiple doctors and specialists responding to patients, the scenario would not have resulted in the patient’s death, whereas a single doctor at a small facility might have done nothing more than suture the wound without fully examining the patient.

“The matter also relates to the level of hospitals and the quality of doctors. A different hospital level has a different clinical quality, and different doctors have different skill levels within the same hospital.” [CD9]

“I think it depends on the hospital, whether it was a one-person hospital or a team hospital, whether the patient was in an intensive care unit or a general ward, and so on. I think there are many different situations.” [JD16]

### Doctors Felt Regret About the Young Patient’s Death

#### I Would Feel Regret for the Patient’s Death (CD and JD)

Most doctors from both countries felt that the patient’s death was regrettable and sad.

“Death is indeed a pity.” [CD5]

“The only feeling is regret.” [JD1]

#### Both the Doctor and Patient were Unlucky (CD)

Several Chinese doctors indicated that both the doctor and patient were unlucky. The patient unfortunately lost his life and his parents were also unlucky to have lost their son. The doctor was also unlucky because the situation is likely to escalate into a serious medical dispute. In addition, a patient seriously injured from a fight is likely to have a poor attitude when receiving medical care, putting the doctor at a disadvantage from the outset.

“Both the doctor and patient are very unlucky in this scenario. The patient paid the price with his life because of his bad behavior, and the doctor will also face many problems due to the sudden death of the patient.” [CD16]

#### The More you Experience Sudden Death Scenarios, the More Timid you become (CD)

One Chinese doctor expressed the sentiment that the longer he works as a doctor and treats more patients who suddenly die, the more timid he becomes.

“If you experience some sudden death cases, you will become more and more afraid.” [CD19]

### There was a Disagreement concerning Responsibility for the Patient’s Death among Doctors from Both Countries

#### If there was No Medical Error, the Doctor Should be Exempt from a Lawsuit (CD)

Many Chinese doctors believed that if Doctor X had not committed a medical error, he should not be subject to medical litigation. This is because the patient in this scenario was seriously injured by the stab wound, and it would have been difficult to save his life even if the doctors had done their best. Some doctors expressed that patients with cervical stab wounds often cannot be saved and that their lives are in an extremely precarious state. Others believed that medical disputes and lawsuits on the grounds of the patient’s death were unreasonable and unacceptable if the doctor had given a full explanation beforehand and there had been no errors in the treatment process.

“If the doctor has perfected the preoperative examination and postoperative monitoring, the death is an accident, and the dispute is unreasonable.” [CD19]

#### The Fight brought about the Patient’s Death (CD and JD)

Doctors from both countries stated that the fight itself was responsible for the patient’s death. Some said that the fight should not have taken place and that the patient had paid the price for his wrongdoing, while others noted that the person who stabbed the patient in the neck was responsible for the patient’s death. Some voiced the opinion that the patient’s family should blame the culprit who attacked the patient, not the doctor, for the patient’s death. The doctor was viewed as being only partially responsible for the patient’s death.

“The family should blame the person who hurt the patient, not the doctor.” [CD3]

“The family should take action against the person who harmed the patient, but ended up directing their anger at the doctor. I think it is necessary to respond to this.” [JD13]

#### It is Inevitable for the Family to be Angry and Initiate a Lawsuit (CD and JD)

Doctors from both countries noted the inevitability of the bereaved family filing a lawsuit because they believe that Doctor X was responsible for the patient’s death. One Chinese doctor suggested that the family would file a medical lawsuit when a patient dies, as they do not see the death as being inevitable and always believe that there must have been a medical error during the treatment process. Chinese doctors also noted that families tend to think that patients could have been rescued if they received better treatment in a better environment. Japanese doctors predicted that anger and blame towards doctors would inevitably lead to medical lawsuits being filed against them. Doctors from both countries predicted that the doctor and hospital would lose the lawsuit.

“The patient is too young; the family will definitely be outraged.” [CD6]

“Family members will definitely lose control of their emotions because they cannot accept.” [JD13]

#### This Case is Very Complicated and Requires Discussion concerning Responsibility for the Patient’s Death (CD and JD)

Some doctors from both countries noted that primary responsibility for the patient’s death was not easy to determine and requires careful consideration, including an autopsy. It was noted that whether or not the doctor had done their best, what had happened during the treatment process, what was the ultimate cause of death, and what was the evidence for it had to be comprehensively scrutinized.

“This scenario is very complicated and requires discussion concerning responsibility for the patient’s death.” [CD1]

“Let’s investigate the cause of death.” [JD5]

### Participating Doctors decided how to Behave after Anticipating the Reaction of the Bereaved Family and their Initial Responses Varied Widely between the Two Countries

Among the results related to the fourth and fifth categories in Table 3, Table 4 provides a summary of representative statements from Chinese and Japanese doctors to exemplify differences in their views on communication with and apologies to the bereaved families (Table 4).

**Table 4:** Examples of doctors’ responses to the hypothetical case

Subcategories selected from Table 3	Representative statements
It is impossible for doctors to communicate with the family due to the possibility of physical violence from bereaved family members (subcategory generated solely from Chinese doctors’ responses)	**(CD6)** *“In order to avoid violent conflict, the doctor must not face the family members directly.”*
	**(CD9)** *“The bad ending is not intentional. But I was afraid that his family members would beat me, so it was better to hide for a while.”*
	**(CD10)** *“Errors like this should be admitted to, but if your honest disclosure would result in the family’s violence, you can’t admit to it.”*
I need to communicate with the family because they would find me quickly (subcategory generated solely from Chinese doctors’ responses)	**(CD7)** *“I am sure that the family members will also come to me first. So, the doctor is the first person to communicate with the family.”*
The first major premise is to properly explain what happened to the family (subcategory generated solely from Japanese doctors’ responses)	**(JD20)** *“If it were me, I would just honestly say what I was thinking in my head at the time, without hiding.”*
	**(JD5)** *“I think I would have no choice but to explain to the family. It is inevitable. I would listen to their opinions and then provide my explanation.”*
It depends on the situation such as the treatment process, characteristics of the patient’s family, and hospital setting (subcategory generated solely from Japanese doctors’ responses)	**(JD16)** *“I would be a bit biased towards a patient who came in after a fight. If the family was an average family, I would give them an explanation in a straightforward way.”*
	**(JD19)** *“Responses to the family may differ slightly in different hospitals. Some hospitals may instruct you to never say sorry.”*
The magnitude of the medical error changes doctors’ attitudes towards honest disclosure (subcategory generated solely from Chinese doctors’ responses)	**(CD13)** *“In general, some small remediable or insignificant errors can be admitted to. Doctors can admit to some errors that can be corrected, but only if they (the patient/patient’s family) are fully informed before treatment. Otherwise, the patient would become angry and might hit you.”*
	**(CD12)** *“Small mistakes are easy to remedy, but big mistakes must be admitted to because the family will find out about it in the end. That would then complicate things more.”*
If there was a medical error, it should be explained to the family as soon as possible to avoid a situation where the family suspects the hospital is hiding the error (subcategory generated solely from Japanese doctors’ responses)	**(JD17)** *“If there is a medical error, we should explain it to the family as early as possible. If we attempt to hide it, we will fall into a mud pit. Not explaining, treating patients rudely, or attempting to conceal errors would increase patient distrust.”*
	**(JD14)** *“If the loss was due to medical negligence of my own, I would make a proper apology.”*
	**(JD16)** *“If a hospital hides from patients and their families that negligence has occurred in patient care, the situation would often be more difficult later than if the hospital honestly tells them that negligence occurred up front.”*

*CD*, Chinese doctor; *JD*, Japanese doctor

#### I would have Reported to the Patient Safety Department of my Hospital First when the Patient Died (CD and JD)

Doctors from both countries noted that following the patient’s death, they would first report the incident to the patient safety officer. They would then deal with the situation in accordance with the normal procedures adopted when a patient unexpectedly dies in a hospital, or a medical dispute is anticipated. Some Japanese doctors said that they would never deal with such situations alone.

“In this scenario, it could only be handled according to the normal procedures for handling disputes.” [CD14].

“I would report this to the hospital management and share the information. The whole hospital needs to respond to the situation.” [JD19].

#### It Is Impossible for Doctors to Communicate with the Family due to the Possibility of Physical Violence from Bereaved Family Members (CD)

Most Chinese doctors noted that it is impossible for them to communicate with the bereaved family unless their own physical safety is guaranteed. The patient’s family members could resort to violence against the doctor from anger. As a result, doctors often are unable to communicate with the patient’s family. Chinese doctors also considered communication with and providing an explanation to the patient’s family useless. This is because even if they communicate with and provide explanations, the bereaved family no longer trusts the doctor. In cases for which a medical error clearly occurred, the doctor has no excuse and can only remain silent. Finally, face-to-face communications between the doctor and the family could lead to an escalation of doctor-patient conflicts. Even if the doctor admits to the error and apologizes for it, this would not resolve the conflict, but only intensify it. One Chinese doctor noted the urgency of needing to find a way to deal with tense situations because the hospital fails to take responsibility in handling disputes, leaving doctors to defend themselves against families.

“In order to avoid violent conflict, the doctor must not face the family members directly.” [CD6]

“The bad ending is not intentional. But I was afraid that his family members would beat me, so it was better to hide for a while.” [CD9]

“Errors like this should be admitted to, but if your honest disclosure would result in the family’s violence, you can’t admit to it.” [CD10]

#### I Need to Communicate with the Family because they would Find Me Quickly (CD)

One Chinese doctor indicated that in cases such as the hypothetical case, doctors usually have no choice but to communicate with the patient’s family first because the bereaved family would be the first to approach the doctor in charge and demand a satisfactory answer. Some doctors noted that even if they hide, the bereaved family would find them quickly with ease. Several Chinese doctors suggested that, if they came face to face with the families, they would first try to appease them.

“I am sure that the family members will also come to me first. So, the doctor is the first person to communicate with the family.” [CD7]

#### The First Major Premise is to Properly Explain what Happened to the Family (JD)

Several Japanese doctors suggested that their basic stance would be to first meet the patient’s family to express their condolences for the patient’s death, but to also explain exactly what happened. Some doctors said that they would join the family in grieving the patient’s death; others indicated they would apologize for what had happened to the patient and that they could not save his life even though they had done their best. It was also noted that doctors need to listen to the opinions of the patient’s family regarding the doctor’s explanation and be sensitive to the bereaved family’s feelings. At the same time, doctors noted that they would be cautious not to overtalk when explaining so as not to be perceived as making excuses. The importance of doctors speaking honestly and without concealment was also suggested.

“If it were me, I would just honestly say what I was thinking in my head at the time, without hiding.” [JD20]

#### It Depends on the Situation such as the Treatment Process, Characteristics of the Patient’s Family, and Hospital Setting (JD)

Several Japanese doctors stated that their attitude towards families after the death of their patient depended on the course of treatment, what kind of people the family members are, and the hospital’s contingency plan. If the bereaved family are anti-social, it is possible that frank explanations would not be given. It was also noted that hospitals in Japan have different policies for dealing with bereaved families in the event of a patient’s unexpected death. It was noted that some facilities would instruct staff doctors never to apologize to the bereaved families.

“I would be a bit biased towards a patient who came in after a fight. If the family was an average family, I would give them an explanation in a straightforward way.” [JD16]

### The Decision on Whether to Disclose an Error and Apologize to the Bereaved Family was made After Careful and Complex Consideration

#### I would Not Admit to My Error to Family Members without an Expert’s Investigation into the Cause of the Patient’s Death to Avoid Unnecessary Trouble (CD and JD)

Doctors from both countries indicated that the presence or absence of medical errors must be objectively determined by experts and that they would not admit to medical errors to family members of the patient based solely on their own judgement. It was noted that the presence or absence of medical errors must not be unilaterally determined by the bereaved family.

“Whether I am right or wrong should not be judged from the patient’s side.” [CD8]

“I cannot admit to errors based on my own judgment.” [JD1]

#### I would Apologize to the Patient’s Family If I Thought that I had made a Medical Error when Providing Patient Care (CD and JD)

Doctors from both countries who were convinced that they had committed a medical error stated that they would acknowledge the medical error and apologize to the bereaved family. Some were of the view that an apology would alleviate their own guilt, while others commented that medical errors cause a feeling of shame. Some doctors said that they would admit to the error and tell the bereaved family that they had done their best, while others said that they would not make any excuses. A Chinese doctor was of the view that patients and their families are wiser these days than they used to be, so medical errors must be admitted to.

“If there is an error, try to remedy it. If it is impossible to remedy, you have to admit to it, but the premise is that you have tried your best.” [CD17]

“But I don’t think that he (Doctor X) did enough. I myself would apologize. I would just say that I had already treated the wound and I thought that was enough. Whatever you say about the situation, the family would think you were trying to make excuses. I don’t make excuses.” [JD2]

#### The Magnitude of the Medical Error Changes Doctors’ Attitudes towards Honest Disclosure (CD)

Some Chinese doctors indicated that serious and apparent medical errors should be admitted to since they cannot stay hidden, and hiding them would only exacerbate the situation concerning medical disputes. However, they also noted that it is not necessary to tell patients and/or their families about a minor and less apparent error that could be corrected and is nonconsequential, because if patients and/or their families find out about such errors, they would blame the doctor. There was also an opinion that a minor error can be admitted to if the medical condition and the possibility of complications were explained well in advance and, in this case, since it was stated, the family should forgive the doctor. Finally, it was noted that serious errors cannot be admitted to because any explanation afterwards would only complicate the situation.

“In general, some small remediable or insignificant errors can be admitted to. Doctors can admit to some errors that can be corrected, but only if they (the patient/patient’s family) are fully informed before treatment. Otherwise, the patient would become angry and might hit you.” [CD13]

#### If there was a Medical Error, It should be Explained to the Family as Soon as Possible to Avoid a Situation where the Family Suspects the Hospital is Hiding the Error (JD)

Some Japanese doctors stated that if the error is discovered, the doctor should disclose the error without any excuses and apologize to the family. This is because timely and honest disclosure could prevent a loss in potential lawsuits since many medical disputes and lawsuits focus on whether the doctor explained or concealed the error. Furthermore, honest and full disclosure can improve the patient’s trust in doctors and the hospital. It was also noted that the manner in which a doctor explains what happened to the family is crucial to avoid patient distrust.

“If there is a medical error, we should explain it to the family as early as possible. If we attempt to hide it, we will fall into a mud pit. Not explaining, treating patients rudely, or attempting to conceal errors would increase patient distrust.” [JD17]

## Discussion

The present study found that both Chinese and Japanese doctors gave similar clinical assessments of the course of care in the hypothetical scenario and had similar emotional reactions to the patient’s death. Doctors from both countries had varying opinions concerning primary responsibility for the patient’s death. However, significant differences were observed between Chinese and Japanese doctors regarding how to communicate with the patient’s bereaved family and whether or not they would admit to and apologize for medical errors. Below, we discuss the ethical and social implications of the similarities and differences between Chinese and Japanese doctors regarding views on responsibility, meeting with the bereaved family, and admitting to medical errors and apologizing from an ethical perspective.

In the sections that follow, we discuss similarities and differences between Chinese and Japanese cultures. However, before doing so, we note in advance the limitations of our cultural comparisons because it would be problematic to attribute the differences in doctors’ responses between the two countries only to cultural differences. Even within the same culture, individuals significantly differ in many ways. The differences in attitudes and ideas of the doctors may have resulted from differences in individual upbringing, life experiences, and discipline at home. Furthermore, different generations of doctors may have different attitudes and behavior patterns. Individuals can also significantly change their cultural perspectives as life progresses. There may also be regional differences within a country. Within the same culture, some aspects change over time while others remain constant. Certain aspects may be common across diverse cultures (Masaki et al. 2014). With this in mind, the following sections consider the thoughts and actions of Chinese and Japanese doctors from a cultural perspective.

### Responsibility for the Patient’s Death

In China, family members tend to view any unexpected adverse events as the responsibility of the doctor and will file a lawsuit regardless of the circumstances (Fu et al. 2020; Li et al. 2022; Xu et al. 2024). Chinese doctors of the present study expressed an explicit and earnest desire for a guarantee that no pursuit of liability would occur in the absence of error. In Japan, medical lawsuits are often filed by the bereaved family to pursue the cause of a patient’s death, and criminal charges of professional manslaughter are occasionally filed. Therefore, doctors from both countries felt that litigation by family members would be inevitable when a young trauma patient dies suddenly, as in the hypothetical scenario (Citizens’ Association for the Disclosure and Publication of Medical Information et al. 2021; Utsuki 2024). It has been pointed out that patients tend to interpret medical errors much more broadly than doctors to include unavoidable complications, poor quality care, and poor communication (Gallagher et al. 2003; Sato 2020; Wakamatsu 2023). Even when doctors provide standard medical care and appropriate explanations, they might be accused of concealing errors or making false statements, which can also lead to lawsuits. This may also increase the anxiety of doctors with regard to litigation risk.

Doctors from both countries agreed that objective and detailed investigations of the causes of a patient’s death are necessary, underscoring the general importance of careful investigation of adverse events and their causes. The difficulty of assigning responsibility and the possibility of disagreement are always present. In particular, as in the present hypothetical scenario, the patient’s death resulted from an injury in a violent incident, and it may have been difficult to save the patient’s life if his condition was too severe to be rescuable. From a broad social perspective, the doctors’ views that the fight itself or those who attacked the patient should be held responsible for the patient’s death are understandable.

### Communication with the Patient’s Family

Actions Chinese and Japanese doctors would take after the patient’s unexpected death include first reporting the incident to the person in charge at the medical facility, suggesting that measures are in place to address unexpected serious adverse events in both countries (Nagao 2019; Zhai 2019; Zhang 2023; Zong et al. 2023). However, attitudes differed greatly between Chinese and Japanese doctors regarding how they would communicate with bereaved families. Many Chinese doctors indicated that they avoid communication with the family due to potential physical danger to themselves. Their perceptions of the DPR are not optimistic since their safety is not guaranteed. Serious workplace violence in China, including doctors being murdered, has been reported and is unlikely to improve any time soon (Jia et al. 2022; Wang and Du 2023; Xu et al. 2024). However, some Chinese doctors noted that they would not run away from the family but instead meet with them at an early stage to appease them. In both scenarios (meeting or not meeting), there is evidently a strong fear of the patient’s family, highlighting the difficult situation that contemporary Chinese doctors face. This fear and anxiety, which can take the form of verbal or physical threats, also applies to Japanese doctors to some extent. Although not as severe a situation as it is in China, harm to doctors from patient violence has also been reported in Japan (Yomiuri Newspaper 2021; Saitama Newspaper 2022; Xu et al. 2024). To ensure the wellbeing of doctors, creating an environment that frees doctors from the anxiety and fear of their patients’ families is important.

The basic attitude of many Japanese doctors when an unexpected adverse event occurs is to explain what happened to the patient’s family, or in the hypothetical scenario, the patient’s bereaved family, without delay, expressing regret for the loss and saying “sorry.” This way of responding is now widely and strongly recommended in medical education in Japan as the basic attitude doctors and health professionals should take when an unexpected adverse event occurs (Sato 2020). Importantly, however, the Japanese expression for “sorry” (*sumimasen*) is polysemic and does not necessarily mean admitting to one’s errors and compensating the other party. Rather, it is often an expression of understanding that one is offended, although whether one takes responsibility and provides compensation is different matter (Senda 2009). Being sorry for what has happened is an expression of empathy and condolence and should be distinguished from an apology that acknowledges errors and responsibility for the occurrence of an adverse event (Nagai 2019). This point is in line with the “Sorry Works!” movement introduced in the United States (Furuta 2023). In a recent Japanese survey, about 20% of Japanese doctors said that they would first say “*sumimasen*” if the patient was harmed, regardless of whether the medical professional was negligent or not (Hashimoto 2023). This is consistent with the attitudes of the Japanese doctors of the present study.

In China, on the other hand, apologizing sincerely and presenting an actual solution (compensation) are considered more important than the act of apologizing itself (Chen 2020). In the Chinese judicial system, an apology is a kind of psychological consolation mainly applied to violations of honor rights, name rights, portrait rights, and legal responsibility rights (Guo and Xie 2020). Thus, the purpose of an apology is to alleviate the patient’s mental harm. In China, almost all medical errors are disclosed by courts or third-party mediators; medical institutions and doctors rarely disclose medical errors on their own initiative (Gao et al. 2015; Guo and Xie 2020). As a result, opportunities for Chinese doctors to apologize directly to patients’ families are also rare. As discussed in the next section, an apology must be considered from various perspectives, including cultural/historical, ethical, legal, and social perspectives.

### Acknowledging Medical Errors and Apologizing

As shown in Table 3, most Chinese and Japanese doctors perceived Doctor X as having made a medical error which resulted in the patient’s death. There were both common and differing attitudes towards error disclosure and apologizing among the doctors. One common point was related to responsibility. They noted that responsibility for the patient’s death should be professionally scrutinized, and no apology or acknowledgment of responsibility should be made in a careless and hasty manner since a medical lawsuit would be an enormous burden (Huang and Dong 2022; Ishiguro et al. 2022).

Consistent with the results from several Western studies, the type of medical error impacts the attitude of Chinese doctors towards error disclosure (Gallagher et al. 2006; Gibelli et al. 2022). The standard approach is to say nothing to the patient unless there are serious long-term consequences and the decision to disclose is still at the doctor’s discretion (Gao et al. 2015). Human psychology may play a role in keeping what can be hidden/unnoticed secret to avoid trouble that can be avoided. Although none of the Japanese doctors made similar comments, there are currently many cases in Japan in which doctors or medical care providers are suspected of concealing medical errors and are being sued (Citizens’ Association for the Disclosure and Publication of Medical Information et al. 2021; Utsuki 2024).

Several factors may cause doctors to hesitate in disclosing medical errors. These include persistence of the traditional culture of secrecy and denial; fear of legal liability, punitive measures, damage to reputation, and loss of patient’s trust; doctor’s incorrect assumption that the error will not leak out and the patient will never find out what happened; the expectation that doctors will always act without error, developing a sense of infallibility; and shame, painfulness of disclosure, and loss of self-image (Lazare 2006; Detsky et al. 2013; Ceriani-Cernadas 2017; Mendonca et al. 2019). On the other hand, major causes of litigation include intentional concealment, lack of empathy, and dishonest explanation of medical errors on the part of doctors and hospitals (Ceriani-Cernadas 2017; Citizens’ Association for the Disclosure and Publication of Medical Information et al. 2021; Ishiguro et al. 2022; Utsuki 2024). What Japanese patients seek in medical litigation is an explanation of what caused the bad outcome, an apology, clarification of legal responsibility, monetary compensation, presentation of measures to prevent recurrence, and pursuit of social responsibility. While the first two motives are universal, the more important motive is to pursue the cause of the bad outcome (Ishiguro et al. 2022). The Chinese public similarly desired formal disclosure of medical errors (Gu and Deng 2021).

When an adverse event occurs due to a medical error, admitting to the error and sincerely apologizing can have a positive impact on maintaining the trust of the patient and the patient’s family and preventing subsequent medical litigation. An apology also leads to forgiveness by the victim. Some families who have lost loved ones in medical accidents noted that, as long as it is clear that the best medical care was provided, there is no need to initiate a dispute even if the patient dies as a result (Citizens’ Association for the Disclosure and Publication of Medical Information et al. 2021).

In our view, the failure of doctors to disclose medical errors is not only contrary to ethical principles but also places them at a further disadvantage. Ethical reasons for doctors to disclose errors include respect for people, respect for the patient’s right to know, the importance of public trust in doctors, professional ethics to protect patient interests, respect for the patient’s right to informed consent, the duty of candor, and the obligation to improve patient safety (Hebert et al. 2001; Jonsen et al 2015; Bourke and Lochtenberg 2022). We agree that patients have the right to know about medical errors and doctors have a duty to disclose them. Doctors are also denying themselves the chance to avoid medical litigation by failing to disclose errors. Doctors should be made fully aware of the ethical importance and consequential utility of error disclosure, and healthcare facilities must remove barriers to realize this.

Apologizing, like medical error disclosure, was found to be a difficult issue for doctors of both countries. Doctors expressed a desire to apologize to their patients if it was clear to them that they made an error. However, this desire was accompanied by different feelings and objectives, such as a sincere desire to apologize or to alleviate guilt. Moreover, the content of what is mentioned during the apology differed. A difference was also noted in the spontaneity of the apology. In general, an apology consists of the following components: acknowledgement of the offence; explanation for committing the offence; expression of remorse, shame, forbearance (a commitment not to repeat the offence), and humility; and reparation (Lazare 2006). An explicit reference to one’s own responsibility can be added to this list (Kawasaki 2018). Generally, these elements must be present to be considered a full and sufficient apology (Kawasaki 2018). Unfortunately, however, there has traditionally been a reluctance to offer apologies in healthcare settings when things go wrong (McLennan et al. 2014).

The present study did not reveal doctors’ primary motivation for apologizing. Multiple thoughts are often on one’s mind when performing an action, and motives for making an apology may neither be simple nor pure. Genuine and sincere apologies and strategic apologies as skills are likely to be mixed (Furuta 2023). From an ethical perspective, however, when an error has been clearly made, making an apology that includes all of the aforementioned components voluntarily and proactively with the primary goal of reducing patient harm is important. While one could hope that the apology would decrease the likelihood of a medical dispute and medical litigation, it would be unprofessional and unethical to have this outcome as the primary goal from the outset.

Apologies are viewed differently in Chinese and Japanese culture. In Japan, the emphasis is on the act of apologizing, beginning with words of apology. If only compensation is provided without an accompanying apology, the person would be considered insincere, and the situation may not resolve. On the other hand, in China, where more importance is placed on compensation, a verbal apology alone may be equated as not having apologized at all (Takahashi 2012). These cultural differences are reflected in the different attitudes towards apologies between Chinese and Japanese doctors, as with the disclosure of medical errors. And we should avoid harming patients and patient families by apologies that ignores cultural differences and the other party’s psychological acceptance.

This study has several limitations. First, differences in background between doctors from both countries (e.g., hospital type and age, as reflected in Table 2) may have affected the validity of our comparisons. The validity of the results could have been increased if it was possible to have recruited study participants with more similar backgrounds. Qualitative studies using sampling methods that match the backgrounds of study participants in both countries or large cross-sectional quantitative comparative studies using random sampling methods will be needed to confirm the validity and generalizability of our results. However, the findings would nonetheless be informative if doctors from both countries shared common experiences of disputes or if they suffer from similar mental health conditions, regardless of the type of hospitals to which they belong.

Second, interviews were conducted in Chinese or Japanese, and then analyzed and presented in English. This process could have introduced translation issues, such as misunderstanding of the details and/or true meaning of the doctors’ comments. However, given the fluency of some of the researchers in Chinese, Japanese, and English, we were able to confirm the quality and authenticity of the English translation.

Finally, although the present study was qualitative and exploratory in nature, only limited generalizations can be made based on our results. Especially, only one scenario was presented to the participating doctors. Different scenarios may have led to different doctor responses. In addition, the scenario used was hypothetical and this could have made it difficult to ascertain how physicians in each country would actually respond to an unexpected death. Social desirability bias might also result in an overestimation of doctors’ willingness to disclose errors and apologize (Gallagher et al. 2006). Nonetheless, our results provide insight into the general tendencies of Chinese and Japanese doctors on this topic.

## Conclusion

Responses to our hypothetical scenario shed light on doctors’ thoughts and actions when an unexpected serious adverse event occurs to a patient. Our findings reveal that responses differ between Chinese and Japanese doctors, and even from individual to individual within the same country. However, there were also common psychological patterns and concerns. We believe it important for doctors to accept responsibility, disclose errors, and sincerely apologize when adverse events occur due to medical errors. Changes to medical practice and society, as well to the psychology and behavior of doctors, will be needed if this is not currently being practiced.

As with medical error disclosure, the act of apologizing may bestow several positive psycho-physiological effects for those harmed, as apologies have the power to heal (Lazare 2006; McLennan et al. 2014). An apology can promote forgiveness, reduce negative effects, and assist in recovery by redressing a power imbalance, restoring dignity, achieving closure, and stopping the search for an explanation or information. An apology can also decrease blame, reduce anger, increase trust, and improve relationships (ACSQHC 2012; Robbennolt 2009; Furuta 2023). Injustice can also be corrected and justice restored with an apology (Furuta 2023). However, there is also the view that the response of those who are apologized to cannot be predicted with certainty and its consequences are uncertain, and it is somewhat of a gamble (Kawasaki 2018; Furuta 2023). From an ethical perspective, regardless of the expected effects of the apology, we consider it important for doctors to provide a sincere apology to patients when a medical error occurs, including recognizing that harm has occurred, attributing responsibility to oneself, expressing regret and remorse, atoning to the victim, and promising that one will never make the same error again in the future (Kawasaki 2018; Furuta 2023).

Educating doctors about the ethics of medical error disclosure and the importance of apologizing likely is insufficient to result in an actual shift in behavior such that doctors would now spontaneously apologize, particularly so without addressing their work overload, inadequacy of the medical system, violence in the medical field, and inevitable nature of medical errors that occur at a certain rate. Barriers to apologizing, such as patients and their families becoming enraged by the apology itself, violence against doctors upon disclosing errors and apologizing, and the use of apologies in legal proceedings (Chen 2020), must be undone. In Japan, if an individual doctor, of their own discretion, admits to an error and prematurely apologizes, there is a high risk that the doctor will be considered to have accepted responsibility in court (Yoshimura et al. 2022). The same concern applies in China (Guo and Xie 2020).

In our view, the following changes are needed. The public should be reminded that errors can occur even when doctors do their best. It must be recognized that complications and medical errors are two different things. In China, the problem of violence against doctors must be addressed. It also is imperative that medical institutions provide adequate psychological support to doctors who make medical errors. The culture of blaming only the individual who made the error, as well as the legal system in which a sincere apology can be legally disadvantageous, must be reconsidered.

Once the barriers are eliminated, doctors should readily apologize to patients for their errors. If doctors voluntarily choose to continue as a medical doctor whose primary role is to serve the best interest of patients and their families, they will be compelled to apologize when errors result in harm to their patients. In contrast, doctors who pursue only self-interest, are busy covering up their errors, and do not feel any guilt even when patients are harmed by their errors are not suited for the profession to begin with. Forced apologies and superficial, from-the-manual apologies are meaningless from an ethical standpoint. We stress the importance of realizing a clinical environment where conscientious doctors need not hesitate to admit medical errors and can willingly apologize to those who have been harmed.

Finally, reasons for not wanting to apologize are unlikely to differ substantially from those for wanting to avoid error disclosure, including deteriorating relationships with patients, being sued, psychological distress including shame and embarrassment caused by the act of apologizing, belief that nothing wrong has been done, damage to a high self-image, loss of respect and trust from others, being misunderstood and making things worse, emotional blame from the patient side, and post-apology reparations and sanctions (Kawasaki 2018). While some of these barriers can and should be removed or alleviated through improvements to the environment and institutional support, there are other barriers that doctors, as professionals who deal with human life, must accept and overcome themselves. Since all of us humans make errors, the pride of being infallible should be abandoned, as it prevents honest apologies.

## Data Availability

The datasets used by and/or analyzed during the study are available from the corresponding author upon reasonable request.
